# Identification and Characterization of MicroRNAs in the Leaf of Ma Bamboo (*Dendrocalamus latiflorus*) by Deep Sequencing

**DOI:** 10.1371/journal.pone.0078755

**Published:** 2013-10-21

**Authors:** Hansheng Zhao, Dongliang Chen, Zhenhua Peng, Lili Wang, Zhimin Gao

**Affiliations:** 1 State Forestry Administration Key Open Laboratory on the Science and Technology of Bamboo and Rattan, International Center for Bamboo, Rattan, Beijing, China; 2 Research Institute of Forestry, Chinese Academy of Forestry, Beijing, China; CSIR-Central Drug Research Institute, India

## Abstract

MicroRNAs (miRNAs), a class of non-coding small endogenous RNAs of approximately 22 nucleotides, regulate gene expression at the post-transcriptional levels by targeting mRNAs for degradation or by inhibiting protein translation. Thousands of miRNAs have been identified in many species. However, there is no information available concerning miRNAs in ma bamboo (*Dendrocalamus latiflorus*), one of the most important non-timber forest products, which has essential ecological roles in forests. To identify miRNAs in *D. latiflorus*, a small RNA library was constructed from leaf tissues. Using next generation high-throughput sequencing technology and bioinformatics analysis, we obtained 11,513,607 raw sequence reads and identified 84 conserved miRNAs (54 mature miRNAs and 30 star miRNAs) belonging to 17 families, and 81 novel miRNAs (76 mature miRNAs and five star miRNAs) in *D. latiflorus*. One hundred and sixty-two potential targets were identified for the 81 novel bamboo miRNAs. Several targets for the novel miRNAs are transcription factors that play important roles in plant development. Among the novel miRNAs, 30 were selected and their expression profiles in response to different light conditions were validated by qRT-PCR. This study provides the first large-scale cloning and characterization of miRNAs in *D. latiflorus*. Eighty-four conserved and 81 novel miRNAs were identified in *D. latiflorus*. Our results present a broad survey of bamboo miRNAs based on experimental and bioinformatics analysis. Although it will be necessary to validate the functions of miRNAs by further experimental research, these results represent a starting point for future research on *D. latiflorus* and related species.

## Introduction

MicroRNAs (miRNAs), initially discovered in *Caenorhabditis elegans* [1], are non-coding RNAs of approximately 22 nucleotides, which exist widely in animals, plants and some viruses [2-4]. MiRNAs have important functions in different developmental stages and metabolic process by mediating gene silencing at transcriptional and post-transcriptional levels [5-7]. In plants, miRNAs regulate leaf morphogenesis, the development of root and flowers and other key processes, such as response to biotic and abiotic stresses, via interactions with their specific target mRNAs [4,8-12].

With the development of next generation sequencing technologies, deep sequencing has become a valuable, powerful and effective high-throughput strategy for identifying novel miRNAs. To date, 21,264 entries representing hairpin precursor miRNAs, expressing 25,141 miRNAs, have been identified in 193 species; moreover, these data are available in the public miRNA database miRBase (Release 19, August 2012) [13]. Hundreds of miRNAs have been identified from *Arabidopsis*, *Brassica rapa*, barley, grapevine, maize, peanuts, rice, wheat, and other plants in previous research [14-25]. However, few studies have focused on the characterization of miRNAs in bamboo, which is one of the largest members of the grass subfamily *Bambusoideae*, of the family *Poaceae* (*Gramineae*) [26].

Bamboo, one of the most important non-timber forest products among the world’s plant and forest resources, is widely distributed in the tropical and subtropical areas under fluctuating light conditions. Most bamboos are fast growing, reaching their full height and diameter within a single growth season, which indicates that bamboos may possess unique carbon assimilation mechanisms in their leaves. *D. latiflorus* is an evergreen species locally known as ‘tropical giant bamboo’, which forms abundant forests in southern China and southeast Asia, and is a valuable natural resource used as food, building material and other human consumption [27]. Consequently, *D*. *latiflorus* is an obvious choice for an initial study of miRNAs in bamboo. 

Using high-throughput sequencing and bioinformatics analysis, we identified 84 conserved miRNAs belonging to 17 families, and 81 novel miRNAs from more than 11 million raw sequence reads generated from a small RNA library of ma bamboo leaf. One hundred and sixty-two potential targets were identified for the 81 novel bamboo miRNAs. In addition, to confirm the novel predicted miRNAs in bamboo leaf tissues, the expression profiles of 30 novel miRNAs under different light conditions were validated by qRT-PCR. These results will lay the foundation for understanding miRNA-based regulation during *D. latiflorus* development. 

## Materials and Methods

### Plant material, RNA isolation and small RNA high-throughput sequencing

Cutting seedlings of ma bamboo (*D. latiflorus*) were potted in our laboratory under a regime of 16 h light and 8 h darkness at 25°C, with a light intensity of 200 μmol·m^-2^·s^-1^ and a relative humidity of 75%. As experimental materials, we chose the third piece of new functional leaf (blade tissue only) from the top of the branch, which could be considered a juvenile leaf. The leaves were collected from 2-year-old cuttings and quickly frozen in liquid nitrogen. Total RNA was isolated from leaf tissues using the Trizol reagent (Invitrogen, Carlsbad, CA, USA), according to the manufacturer’s instructions.

The small RNA library construction for ma bamboo and Solexa sequencing were carried out at BGI-Shenzhen (Shenzhen, China) using the standard Solexa protocol [28]. Briefly, small RNAs of 15–30 nt in length were first isolated from the total RNA through 15% TBE urea denaturing polyacrylamide gels. Subsequently, 5′ and 3′ RNA adaptors were ligated to these small RNAs, followed by reverse transcription into cDNAs. These cDNAs were amplified by PCR and subjected to Solexa sequencing. After removing low quality reads and trimming adapter sequences, small RNAs ranging from 18–30 nt were collected and used for further analyses. Finally, the selected clean reads were analyzed by BLAST against the Rfam database (http://rfam.sanger.ac.uk/) [29] and the GenBank non-coding RNA database (http://www.ncbi.nlm.nih.gov/) to discard rRNA, tRNA, snRNA and other ncRNA sequences. In addition, sequencing data within this study were firstly uploaded to NIH Short Read Archive (accession number: SRX347876).

### Data content

To obtain and analyze miRNAs from the leaf of ma bamboo, three types of data were used in this study (1). MiRNA data. The miRNA data from the leaf of ma bamboo was obtained by next generation sequencing, and all the reference sequences of mature miRNAs, star miRNAs and their precursors (pre-miRNAs) were downloaded from miRBase, Release 19.0 (http://www.mirbase.org/index.shtml) [13]. (2) Genome data of moso bamboo (*Phyllostachys heterocycla* *var.* *pubescens*) [30]. Although the whole genome sequence of ma bamboo is not available, phylogenomic analyses suggested that ma bamboo and moso bamboo had the closest relationship, with high sequence similarity [31]. For this reason, the sequenced genome of moso bamboo from our previous study was used as the genome to analyze ma bamboo miRNA data (3). Expressed sequence tag (EST) and mRNA data from ma bamboo. The approach outlined in (2) would inevitably produce some biases and some miRNAs would not be identified. Therefore, to fill the gap, ESTs and other mRNA data from ma bamboo were obtained from the NCBI database site and used to identify additional miRNAs.

### Prediction of conserved miRNAs, novel miRNAs and potential miRNA targets in ma bamboo

As miRNA precursors have a characteristic fold-back structure, 150 nt of the sequence flanking the genomic sequences of small RNAs (sRNAs) was extracted and used to predict miRNAs. For analysis of conserved miRNAs in ma bamboo, unique sRNAs were aligned with plant mature miRNAs in miRBase Release 19.0. First, after rigorous screening, all retained sequences with three or more copies were considered as potential miRNAs. Second, sRNA sequences with no more than four mismatched bases were selected by BLAST searching against miRBase. Third, the remaining 15–26 nt reads were used to map the genome of moso bamboo and ESTs of ma bamboo using the BLASTN program. Sequences with a tolerance of two mismatches were retained for miRNA prediction. RNAfold (http://www.tbi.univie.ac.at/RNA/) [32] was used for secondary structure prediction (hairpin prediction) of individual mapped miRNAs, using the default folding conditions to identify the known conserved miRNAs in ma bamboo. Finally, sequences that were not identical to the conserved miRNAs were termed novel miRNAs. 

Potential target sequences for the newly identified miRNAs were predicted using the psRNATarget program (http://plantgrn.noble.org/psRNATarget/) with default parameters. Newly identified miRNA sequences for ma bamboo were used as custom miRNA sequences, while coding sequences for moso bamboo, as well as EST and mRNA databases for ma bamboo, were used as custom plant databases, respectively. All predicted target genes were evaluated by the scoring system and criteria defined in a previous report [33]. Sequences with a total score less than 3.0 were identified as miRNA targets.

### Expression analysis of novel miRNAs by qRT-PCR

The stem-loop qRT-PCR method [3] was used to detect the expression levels of novel miRNAs in *D. latiflorus*. Forward primers were specifically designed for each individual miRNA, as detailed in a previous method [3]: six nucleotides of the 3' end of the stem-loop RT primer were complementary to the 3' end of the mature miRNA, and the sequence 5' - CTCAACTGGTGTCGTGGAGTC - 3' was used as the universal reverse primer [34]. U6 snRNA was used as an internal control [35]. More detailed information is supplied in File S1.

Total RNA was isolated from leaves under three different light conditions, namely CK (the control of 200 μmol•m^-2^•s^-1^ for 4 h), high light (1200 μmol•m^-2^•s^-1^ for 4 h) and dark (dark for 24 h). cDNAs were synthesized from total RNA with the miRNA-specific stem-loop RT primer. The volume of the reverse transcription reaction was 15 μL, which contained 300–500 ng total RNA, 1 μL (2 μM) of RT primer, 3 μL of 5 × reaction buffer, 1.3 μL (each 10 mM) dNTP mix, 1 μL (40 U/μL) RNAsafe and 0.5 μL (200 U/μL) M-MLV reverse transcriptase. The reaction was incubated at 16°C for 30 min, 42°C for 30 min, 85°C for 5 min and 4°C for 5 min. 

The qRT-PCR was conducted using a SYBR Green I Master Kit (Roche, Germany) on a LightCycler^®^ 480 Real-Time PCR System (Roche). The final volume was 20 μl, containing 7.5 μl 2×SYBR Premix Ex Taq, 0.3 μl of each primer (10 μM), 2 μl of cDNA and 7.2 μl of nuclease-free water. The amplification was carried out as follows: initial denaturation at 95°C for 10 min, followed by 41 cycles at 95°C for 10 s, 55°C for 20 s, and 72°C for 10 s. The melting curves were adjusted as 95°C for 5 s and 55°C for 1 min and then cooled to 40°C for 30 s [36]. All reactions were repeated three times.

For each condition, the qRT-PCR experiments were performed as biological triplicates and expression levels were normalized according to that of the internal control. The relative value of the gene expression was calculated using the 2^-ΔΔCt^ method [37]. Statistical tests were performed on the qRT-PCR data using SPSS (Statistical Product and Service Solutions) 18.0 software. Error bars representing the standard deviation were derived from the three experiments in triplicate.

## Results and Discussion

### Overview of small RNA library sequencing

Deep sequencing of the small RNA library from *D. latiflorus* leaf tissues produced 11,513,607 raw sequence reads. Low quality sequences, adapters and small sequences shorter than 16 nucleotides (nt) were removed, leaving 10,593,305 clean reads and 6,320,379 unique sequences. After further removal of unannotated small RNAs and non-coding RNAs, such as tRNAs, rRNAs, siRNAs, snRNAs, snoRNAs and other non-coding RNAs, 910,151 miRNA sequences, accounting for 8.59% of the total sRNA, were identified (Table 1). 

**Table 1:** Summary of small RNA in sequencing data.

**Category**	**Unique sRNA**	**Percent (%)**	**Total sRNA**	**Percent (%)**
Total	6320379	100%	10593305	100%
miRNA	16853	0.27%	910151	8.59%
rRNA	30601	0.48%	170619	1.61%
repeat	13908	0.22%	46909	0.44%
siRNA	137281	2.17%	748553	7.07%
snRNA	841	0.01%	1411	0.01%
snoRNA	634	0.01%	859	0.01%
tRNA	4385	0.07%	58977	0.56%
Unannotated	6104298	96.58%	8638842	81.55%

**Figure 1:**
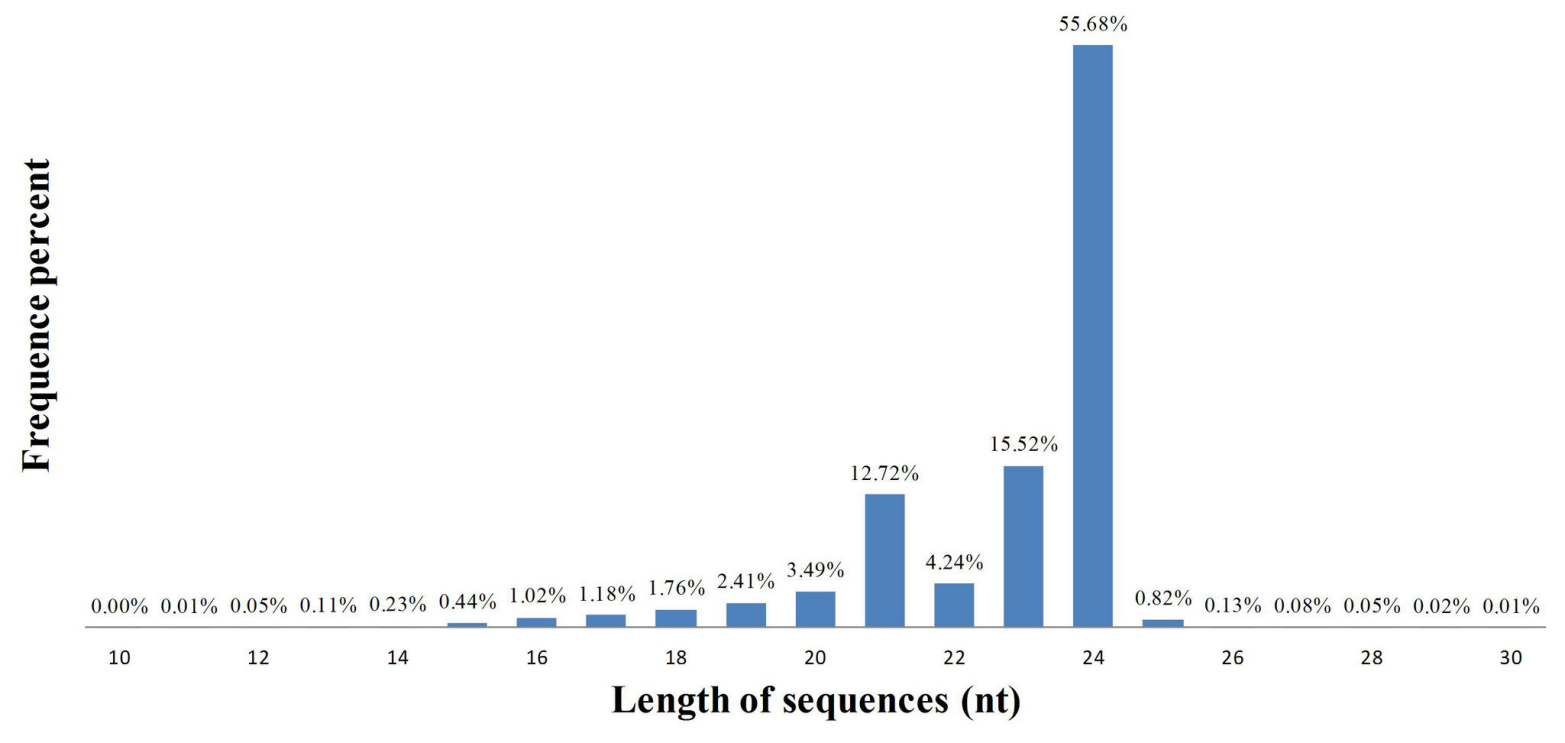
Length, distribution and abundance of small RNAs in *D. latiflorus*.

The significant feature of the size profile permitted miRNAs to be distinguished from other small RNAs. The sRNAs length distribution (10–30 nt) of the original reads demonstrated that the most abundant reads were those of 20–24 nt in length (Figure 1), which were consistent with sRNAs with known function [19]. The most abundant class was those of 24 nt, which was also highly consistent with those small RNA of other *Poaceae* plants based on Solexa sequencing technology, such as rice [38], barley [39] and wheat [40].

### Identification of conserved miRNAs in ma bamboo

Conserved miRNAs have been found in many plant species and play significant roles in plant development and stress responses [4]. To identify the conserved miRNAs in *D. latiflorus*, the sRNA library was searched using BLASTN for unique mature plant miRNA sequences in miRBase Version 19.0, in which miRNAs of bamboo had not been identified. Following a set of strict filtering criteria and further sequence analysis, 54 conserved miRNAs and 30 conserved star miRNAs were identified from *D. latiflorus*, which belonged to 17 families (Table 2). 

**Table 2:** Conserved miRNAs in *D. latiflorus*.

miRNA family	Name	Novel mature miRNA			Precursor		
		Sequence	Read count	Length (nt)	Sequence	Read count	Length (nt)
156/157	dla-miR156a	gcucacuucucuuucugucauc	24	22			
	dla-miR156b-1	ugacagaagagagugagcac	100411	20	gcucgcuccucuuucugucagc	523	22
	dla-miR156b-2	ugacagaagagagugagcac	100415	20			
	dla-miR156b-3	ugacagaagagagugagcac	100411	20			
	dla-miR156b-4	ugacagaagagagugagcac	100411	20			
	dla-miR156b-5	ugacagaagagagugagcac	100297	20			
	dla-miR156b-6	ugacagaagagagugagcac	68524	20	gcucacugcucugucugucauc	60	22
	dla-miR156b-7	ugacagaagagagugagcac	68524	20	gcucacugcucugucugucauc	59	22
	dla-miR157	uugacagaagauagagagcac	8664	21	gcucucuaugcgucugucauc	16	21
160	dla-miR160a-1	gcgugcaaggagccaagcaug	41	21	ugccuggcucccuguaugcca	7	21
	dla-miR160a-2	gcgugcaaggagccaagcaug	41	21	ugccuggcucccuguaugcca	7	21
	dla-miR160a-3	gcgugcaaggagccaagcaug	41	21	ugccuggcucccuguaugcca	7	21
	dla-miR160a-4	gcgugcaaggagccaagcaug	41	21	ugccuggcucccuguaugcca	7	21
164	dla-miR164a	uggagaagcagggcacgugca	3427	21	caugugcccgucuucuccacc	12	21
	dla-miR164b	uggagaaguagggcacaug	40	19			
165/166	dla-miR165a	ucggaccaggcuucauucc	2843	19	gaauguuggcuggcucgaggc	247	21
	dla-miR165b-1	ucggaccaggcuucauucccc	19188	21	gggaauguugucuggcccgaga	4	22
	dla-miR165b-2	ucggaccaggcuucauucccc	18322	21	ggaguguugucugguccgagaccu	3	24
	dla-miR165b-3	ucggaccaggcuucauucccc	18322	21	ggaacguugucugguccgagaccu	3	24
	dla-miR165c	ucggaccaggcuucauuccuc	5615	21	gaauguuggcuggcucgaggc	248	21
	dla-miR166	ucucggaucaggcuucauucc	566	21			
167	dla-miR167a	ggucaugcugcggcagccucacu	13	23			
	dla-miR167b-1	ucagaucaugcugugcaguuucauc	40	25			
	dla-miR167b-2	ucagaucaugcugugcaguuucauc	40	25			
	dla-miR167c-1	ugaagcugccagcaugaucuga	14355	22	ucagaucaugcugugcaguuucauc	40	25
	dla-miR167c-2	ugaagcugccagcaugaucuga	14355	22	ucagaucaugcugugcaguuucauc	40	25
168	dla-miR168-1	ucgcuuggugcagaucgggac	594369	21	cccgccuugcaccaagugaau	375	21
	dla-miR168-2	ucgcuuggugcagaucgggac	594369	21	cccgccuugcaccaagugaau	375	21
169	dla-miR169a	cagccaaggaugacuugccga	211	21	cggcaaguuguucuuggcuac	60	21
	dla-miR169b	uagccaaggaugauuugccu	12	20			
171	dla-miR171a	caugauauuguuucggcucaug	34	22	ugagccgaaccaauaucacuc	6	21
	dla-miR171b	ugauugagccgugccaauauc	169	21	auauuggugcgguucaaucag	12	21
	dla-miR171c-1	uugagccgcgucaauaucucu	187	21	agauauuggcgugccucaguc	11	21
	dla-miR171c-2	uugagccgcgucaauaucucu	187	21	agguauuggcgugccucaauc	11	21
	dla-miR171c-3	uugagccgcgucaauaucucu	187	21			
319	dla-miR319-1	agugaaugaagcgggagguaa	4	21	agcugccgacucauucacc	4	19
	dla-miR319-2	agugaaugaagcgggagguaa	4	21	agcugccgacucauucacc	4	19
390	dla-miR390	aagcucaggagggauagcgcc	2045	21	ugcuaucuauccugagcucc	52	20
396	dla-miR396	uccacaggcuuucuugaacug	3764	21			
399	dla-miR399	ugccgagggagaacugcacug	170	21			
528	dla-miR528-1	uggaaggggcaugcagaggag	27	21			
	dla-miR528-2	uggaaggggcaugcagaggag	27	21			
535	dla-miR535-1	ugacaacgagagagagcacgc	77827	21	gugcccccucccguugucacu	11	21
	dla-miR535-2	ugacaacgagagagagcacgc	77740	21	gugcucccucccguugucacu	62	21
	dla-miR535-3	ugacaacgagagagagcacgc	77740	21	gugcuccuucccguugucacu	62	21
	dla-miR535-4	ugacaacgagagagagcacgc	77740	21	gugcuccuucccguugucacu	58	21
	dla-miR535-5	ugacaacgagagagagcacgc	77740	21			
	dla-miR535-6	ugacaacgagagagagcacgc	77740	21			
827	dla-miR827	uuagaugaccaucagcaaaca	179	21			
1318	dla-miR1318-1	ucaggagagaugacaccgaca	154	21			
	dla-miR1318-2	ucaggagagaugacaccgaca	149	21			
	dla-miR1318-3	ucaggagagaugacaccgaca	132	21			
	dla-miR1318-4	ucaggagagaugacaccgaca	151	21			
1878	dla-miR1878	acuuaaucuggacacuauaaaaga	7	24			

Compared with the conserved miRNA families in plants reported by Cuperus et al [41], 15 conserved miRNA families, accounting for 88% of the conserved miRNA families in ma bamboo, were represented in ma bamboo. However, of the 29 conserved miRNA families in *ehrhartoideae* [41], 14 conserved miRNA families were not represented in ma bamboo. This may have resulted from the data in miRBase database being a different version, may represent the particular evolution and function of miRNAs in ma bamboo, or may have resulted from the inevitable biases in processes such as the preparation of samples, sequencing and analysis. Further experiments using increased amounts of data are required to verify the conserved miRNA families in ma bamboo. In addition, based on the moso bamboo genome, 13 conserved miRNA families were predicted and compared with those experimentally derived from ma bamboo. This analysis indicated that eight conserved miRNA families, accounting for 62% of all families predicted in moso bamboo, were represented in ma bamboo. This may be explained by the fact that they are two different bamboo species with distinct biological and evolutionary features. For example, the number of chromosomes is 68 and 48 in ma bamboo and moso bamboo, respectively. 

**Figure 2:**
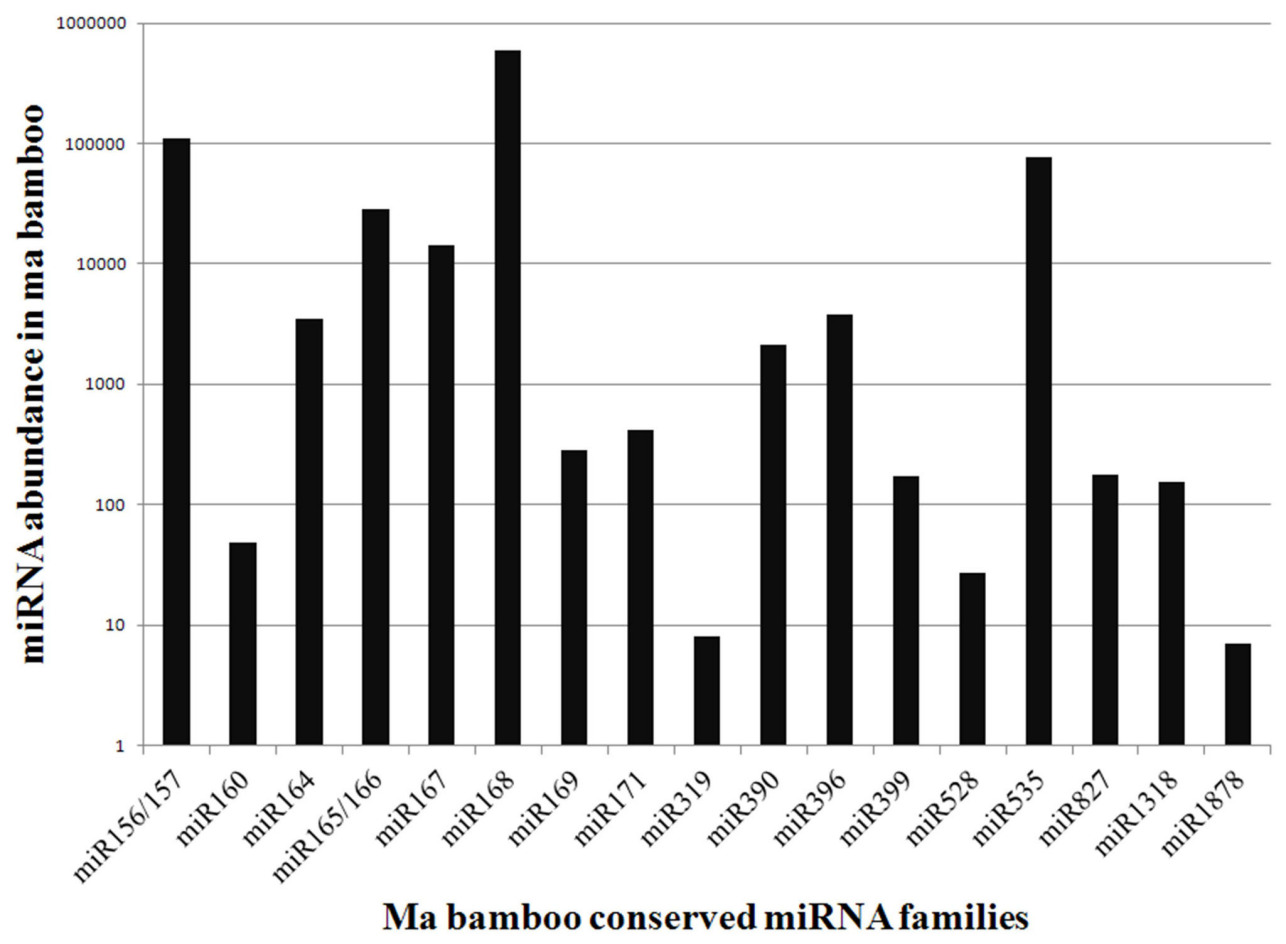
Abundance of conserved miRNA families in *D. latiflorus*.

MiRNAs with high sequencing frequencies have been shown to play fundamental and essential regulatory functions in maintaining biological processes. Therefore, the read counts for known miRNA families were analyzed (Figure 2). The most abundant miRNA family (594,744 reads) was miR168, which was represented five to 41 times more frequently than other relatively high abundance miRNAs, including miR156/157, miR535, miR165/166 and miR167, whose total abundances ranged from 14,448 to 109,638 reads. Moreover, the top three most abundant miRNA family members (miR168, miR156/157 and miR535) were represented by 594,744, 109,642 and 77,838 reads, respectively, accounting for ~93.6% of the expressed miRNA tags. However, the lowest abundant miRNA families (miR528, miR319 and miR1878) represented only ~0.003% of expressed miRNA tags, indicating that these miRNAs were expressed at a low level.

The number of members in the differently conserved miRNA families was also analyzed. Four families (miR156/157, miR165, miR167 and miR535) contained multiple members, with nine, six, five and six members respectively. Five families, miR390, miR396, miR399, miR827 and miR1878, only had one member. 

The bamboo flowering cycle can take up to 120 years and also involves infrequent and unpredictable flowering events, as well as peculiar monocarpic behavior, e.g., flowering once before culm death [26]; therefore, the mechanism of flowering, as a unique characteristic of bamboo, has received much research interest. Studies in other plants indicated that miRNAs play important roles in flower development [42,43]. The transition from juvenile to adult phase is controlled by miR156 and miR172. MiR172 also influences floral organ identity, as evidenced by failure of carpel abortion from the male inflorescence [42]. In addition, extensive analysis of certain monocotyledonous species, such as *Brachypodium distachyon*, *Oryza sativa*, *Sorghum bicolor*, and *Zea mays*, revealed more than three members in each miR172 family. Therefore, absence of miR172 may contribute to bamboo’s special regulatory mechanism of flower development. Other miRNAs (miR164, miR167, miR169 and miR319) with functions during different stages of flower development were detected in *D. latiflorus*.

**Figure 3:**
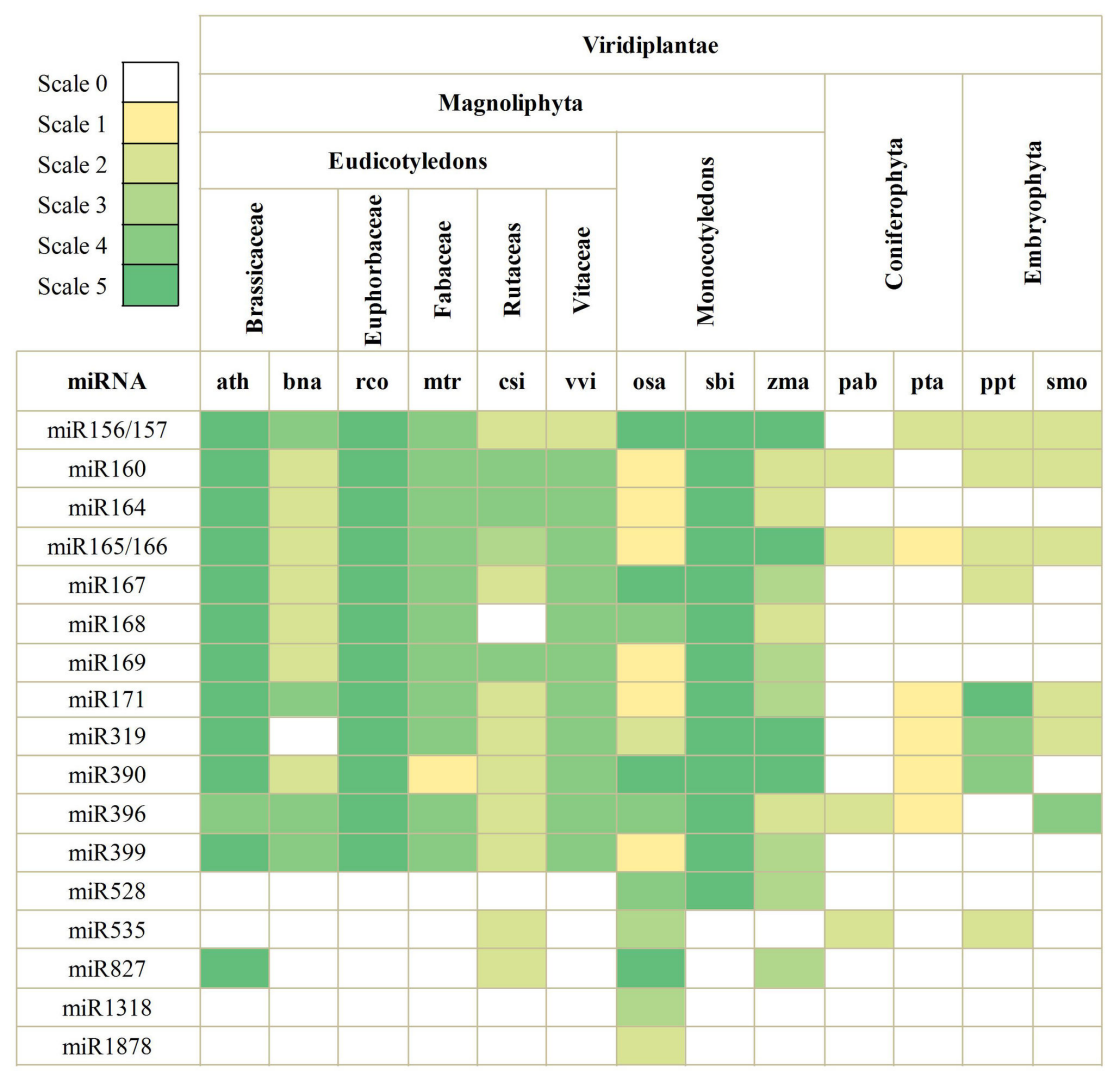
Conserved miRNAs from *D. latiflorus* and their homologs in other some species. Scale 0: miRNA undetected; Scale 1: miRNA identified using computational programs; Scale 2: miRNA sequenced using experimental cloned or high-throught technology; Scale 3: miRNA/miRNA* accumulation detected; Scale 4: miRNA detected by small RNA blot or RT-PCR or Northern; Scale 5: miRNAs with validated targets. Total information in Figure 3 is from miRBase 19.0 and references 14,47,63-84. Abbreviation: ath, *Arabidopsis thaliana*; bna, *Brassica napus*; rco, *Ricinus communis*; mtr, *Medicago truncatula*; csi, *Citrus sinensis*; vvi, *Vitis vinifera*; osa, *Oryza sativa*; sbi, *Sorghum bicolor*; zma, *Zea mays*; pab, *Picea abies*; pta, *Pinus taeda*; ppt, *Physcomitrella patens*; smo, *Selaginella moellendorffii*.

As seen in Figure 3, to explore the evolutionary roles of these conserved miRNAs, deep analyses focused on extensive comparisons against known conserved miRNAs in other plant species, including *Picea abies*, *Pinus taeda*, *Physcomitrella patens*, *Selaginella moellendorffii*, *Arabidopsis thaliana*, *Brassica napus*, *Ricinus communis*, *Medicago truncatula*, *Citrus sinensis*, *Vitis vinifera*, *O. sativa*, S. bicolor and *Z. mays*. Based on BLAST searches and sequences analysis, some miRNA families of *D. latiflorus* (miR156/157, miR160, miR165/166, miR171, miR319, miR390 and miR396) are highly conserved and ancient. For example, miR156/157 is a family of endogenous miRNAs with a relatively high expression level in the juvenile phase of many plants. The level of miR156/157 gradually decreases with plant age [44,45]. 

However, some miRNA families (miR528, miR535, miR827, miR1318 and miR1878) are less evolutionarily conserved and are involved in regulation of diverse physiological processes. For example, the miR528-target recognition site is only present within monocot genes, while all eudicot genes orthologous to *SsCBP1* from *Arabidopsis*, poplar, grape, and soybean genomes completely lack the miR528-target recognition site, which supports the view that miR528 is a monocot-specific miRNA [46]. Another example is miR535, which was predicted to target a gene encoding a brassinosteroid signaling positive regulator protein in *Physocmitrella patens* [47]. It was reported that mature miR535 may be considered as a divergent member of the miR156/157 family, and targeting of *SPL* genes may be a vestigial function of miR535, which may have been performed more efficiently in the course of evolution by other members of the family [48]. 

The remaining five miRNA families, including miR164, miR167, miR168, miR169 and miR399, are homologous in eudicotyledons and monocotyledons, indicating these miRNA families may be recent and are more sensitive to certain stresses. For example, miR168 regulates loop ARGONAUTE1 (AGO1) homeostasis, which is crucial for the regulation of gene expression and plant development [49]. Another report demonstrated that *NFYA5* contained a target site of miR169, which was downregulated by drought stress through an ABA-dependent pathway [50,51]. MiR399 is upregulated in phosphate starvation and its target gene, encoding a ubiquitin-conjugating E2 enzyme, is downregulated in *A. thaliana* [50,51].

### Identification of novel miRNAs in ma bamboo

Previous studies have shown that each species has species-specific miRNAs [17,18,24,25,33,39,52-54]; therefore, ma bamboo is also likely to have unique miRNAs. In addition, a distinct feature of miRNAs is the ability of their pre-miRNA sequences to adopt the canonical stem-loop hairpin structure. After excluding sRNA reads homologous to known miRNAs and other non-coding sRNAs, the remaining 18-24 nt sRNAs were subjected to secondary structure analysis of their precursors using RNA fold software. Seventy-six novel miRNAs and five novel star miRNAs were identified as possible miRNA candidates in ma bamboo (Table 3). ESTs of ma bamboo were used to predict miRNAs; however, no novel miRNAs were detected, which might be explained by the small number of ESTs. 

**Table 3:** Novel miRNAs in *D. latiflorus*.

Name	Novel mature miRNA			Precursor			
	Sequence	Read count	Length (nt)	Precursor ID	Position	Stand	MFE (kcal/mol)
dla-miRC1	ucuugaccuuguaagacccaa	4801	21	PH01001979	131613.131676	-	-22.8
dla-miRC2-1	ccaaccguugggcgacugcau	786	21	PH01004130	67055.67115	-	-34.8
dla-miRC2-2	ccaaccguugggcgacugcau	786	21	PH01002446	47333.47393	-	-38.8
dla-miRC2-3	ccaaccguugggcgacugcau	786	21	PH01005888	46451.46508	+	-26
dla-miRC2-4	ccaaccguugggcgacugcau	762	21	PH01000353	108717.108774	+	-31.3
dla-miRC3	uacucccgggaguagaaaagaggc	654	24	PH01002398	189512.189568	+	-30.2
dla-miRC4-1	ugcuggagaagcagggcacgu	640	21	PH01000210	590922.591000	-	-44.1
dla-miRC4-2	ugcuggagaagcagggcacgu	640	21	PH01000543	161249.161319	+	-34.8
dla-miRC5	gggagagaggaggagagagaaga	375	23	PH01000541	78049.78131	+	-25.1
dla-miRC6	gcuggcuauaggauagauaggaag	312	24	PH01243766	66.129	-	-23.7
dla-miRC7	uggcccuguuuggaaugcgggaau	217	24	PH01001115	391586.391670	-	-32.3
dla-miRC8	gccaagaacguguaagagauggag	210	24	PH01030611	1103.1175	-	-21.5
dla-miRC9	ggguggagcugugccaaacggggc	207	24	PH01000285	221024.221089	-	-30
dla-miRC10	ccauaguagacgccucaucacaga	163	24	PH01004259	20814.20883	-	-27.4
dla-miRC11	acaccugggaguagaaaaaaggcu	150	24	PH01000105	568774.568850	+	-30.3
dla-miRC12	ccaaccauugggcgacugcau	124	21	PH01000070	852964.853021	-	-36.8
dla-miRC13	uuuggaacauaggaauuuca	119	20	PH01006954	13751.13847	+	-23.4
dla-miRC14	gauacuagaauaucacagacgguu	97	24	PH01001207	363449.363523	+	-38.4
dla-miRC15	aggaagagagagaaggaagcg	90	21	PH01002001	139567.139635	-	-27.4
dla-miRC16	gguggagcugugccaaacagg	63	21	PH01003096	129017.129079	+	-34.7
dla-miRC17-1	agguagagcugugccaaacgg	61	21	PH01003063	37258.37318	+	-30.1
dla-miRC17-2	agguagagcugugccaaacgg	61	21	PH01001111	96856.96916	-	-26.4
dla-miRC17-3	agguagagcugugccaaacgg	61	21	PH01003063	37297.37377	+	-26.5
dla-miRC18	cugguucgguccauguagaggugu	59	24	PH01003226	54563.54634	+	-25.8
dla-miRC19	cuuggacugaccaagccacauggu	55	24	PH01001925	2237.2321	-	-21.3
dla-miRC20	uucuagauaagaaccgucugugau	46	24	PH01000405	391719.391798	+	-32.7
dla-miRC21	ggcccugacugcgucgaccucgg	42	23	PH01002012	253987.254051	+	-20.3
dla-miRC22	cgcaggaaucgggaaaaauu	40	20	PH01002694	92485.92570	-	-29.6
dla-miRC23-1	accuaggaccaagagggggaggga	37	24	PH01001705	131691.131777	-	-30.1
dla-miRC23-2	accuaggaccaagagggggaggga	37	24	PH01001705	125497.125583	-	-30.1
dla-miRC24	aacacagacggucucuaaaccaaa	36	24	PH01001117	152549.152626	+	-29.5
dla-miRC25-1	guaugaagguuuguuggcuggcau	34	24	PH01003289	73533.73598	-	-23.8
dla-miRC25-2	guaugaagguuuguuggcuggcau	33	24	PH01001281	374181.374246	+	-22.6
dla-miRC26	gaggcaagugcaugguagaauagu	32	24	PH01001847	191714.191783	-	-24.6
dla-miRC27-5p	gucaucauagccacaaaagaucgu	31	24	PH01001060	7263.7355	+	-44.7
dla-miRC27-3p	gaucuucuguggcuaugaugacau	22	24	PH01001060	7263.7355	+	-44.7
dla-miRC28-1	cacagacgguuguaauuag	31	19	PH01002875	30884.30954	+	-46.1
dla-miRC28-2	cacagacgguuguaauuag	31	19	PH01001151	55189.55261	+	-39.2
dla-miRC29	acuagagcugugccaaacgg	31	20	PH01000462	484606.484666	+	-28.6
dla-miRC30-5p	gacuauguggucguaaaag	31	19	PH01003863	85885.85952	-	-21.3
dla-miRC30-3p	gacuacauggucguagaag	7	19	PH01003863	85885.85952	-	-21.3
dla-miRC31	uucuuaucuagaaccgucugugau	28	24	PH01000334	424366.424425	+	-21.8
dla-miRC32	uguguagauuaucacagacgguu	28	23	PH01001216	399131.399203	+	-26.6
dla-miRC33	gaggaggaggagcagccagcu	27	21	PH01002565	134432.134516	-	-43.1
dla-miRC34	ugagguggucguaggaaag	25	19	PH01000062	595060.595120	-	-27.5
dla-miRC35	guuggaggagagagaagaga	24	20	PH01000059	1124542.1124604	+	-28.2
dla-miRC36	gacuaugugaucguagaag	23	19	PH01004933	41409.41498	-	-39.7
dla-miRC37	cugccaaacaggcccuua	23	18	PH01000905	736.808	-	-28.3
dla-miRC38-1-5p	uugcccaaugguuggacgacu	22	21	PH01003262	22388.22461	-	-38.9
dla-miRC38-1-3p	cgcuccaccguucggcgacu	5	20	PH01003262	22388.22461	-	-38.9
dla-miRC38-2	uugcccaaugguuggacgacu	22	21	PH01000012	523648.523728	+	-37.2
dla-miRC39	guaagggucuguuuggaag	20	19	PH01001784	229612.229696	+	-30.1
dla-miRC40	gaggagauggggaaagug	20	18	PH01000623	330358.330443	+	-28.2
dla-miRC41-1	acggcacggcacgacggaaugacc	19	24	PH01001751	7934.8008	+	-35.9
dla-miRC41-2	acggcacggcacgacggaaugacc	19	24	PH01001751	7880.7958	+	-33.6
dla-miRC42	ccuaagggucuguuugggac	19	20	PH01195637	413.496	+	-31
dla-miRC43	caucuauagacgguacuaaagacg	18	24	PH01002139	217146.217207	-	-22.1
dla-miRC44-1	gacuacguggucguaaaag	17	19	PH01001033	54852.54919	+	-34.8
dla-miRC44-2-5p	gacuacguggucguaaaag	17	19	PH01004351	50225.50292	+	-28.3
dla-miRC44-2-3p	gacuacguggucguagaag	10	19	PH01004351	50225.50292	+	-28.3
dla-miRC44-3	gacuacguggucguaaaag	17	19	PH01000978	326474.326539	+	-23.3
dla-miRC45	ccaaugguugggcgacag	16	18	PH01000015	1202809.1202885	+	-39.9
dla-miRC46	uaggccuaaugccacgucucc	15	21	PH01002414	37289.37354	-	-25.4
dla-miRC47	uuuagggucuguuugggau	15	19	PH01000528	120129.120212	-	-25.3
dla-miRC48	guugaaggugcuaagggaaaggac	14	24	PH01000301	746041.746129	+	-26.4
dla-miRC49	ugggauuaucacagacgguucuu	13	23	PH01003822	73010.73079	+	-30.8
dla-miRC50	guguaucagacgguucuugaugug	13	24	PH01004651	31471.31527	+	-30.5
dla-miRC51	guggaacuggagcuggug	12	18	PH01000859	1107.1183	+	-32.4
dla-miRC52	gauaucuguagauagaguaggagc	11	24	PH01000166	633213.633296	+	-22.3
dla-miRC53	gucguuucucaagugguggaccuc	11	24	PH01001097	143567.143631	+	-21.2
dla-miRC54	cacugacacgugggcuaag	10	19	PH01000466	170309.170382	+	-32.8
dla-miRC55	ucuugaucguugauuucag	10	19	PH01000693	345374.345449	+	-33.9
dla-miRC56	gugauaauuguaacacugacgguu	9	24	PH01001963	79707.79782	+	-35.1
dla-miRC57-5p	guccaaccguuggaugacuuggca	8	24	PH01001235	7375.7464	+	-40.9
dla-miRC57-3p	ccaacgguugagcgacua	6	18	PH01001235	7375.7464	+	-40.9
dla-miRC58	uuaagggucuguuugggau	8	19	PH01000345	528117.528198	+	-41.9
dla-miRC59-1	guucaaccguuggacgauua	7	20	PH01003601	133072.133149	-	-37.6
dla-miRC59-2	guucaaccguuggacgauua	7	20	PH01003390	64643.64719	-	-33.5
dla-miRC60	gucuugugcaguggaccguggugu	7	24	PH01000115	379347.379411	-	-24.9
dla-miRC61	guacuguuggcucagcccaggcac	6	24	PH01000878	437404.437477	+	-39.9
dla-miRC62	agugaaugaugcgggagacu	4	20	PH01000130	143043.143121	-	-37.8

Precursors of these novel miRNAs were identified and formed proper secondary hairpin structures, with minimal folding free energy (MFE) ranging from -20.3 kcal·mol^-1^ to -46.1 kcal·mol^-1^ (average of ~ -31.0 kcal·mol^-1^).

Moreover, the length distributions of plant pre-miRNAs are more heterogeneous than animal pre-miRNAs. The shortest pre-miRNA is 53 nt in length (miRBase ID: ath-MIR5645b) and the longest in 938 nt in length (miRBase ID: aly-MIR858) in miRBASE Version 19. In ma bamboo, the distribution of pre-miRNAs is 54-96 nt, which is consistent with the pre-miRNAs distributions in other plants [17,25,39]. In addition, the length distribution of novel miRNA ranged from 18–24 nt in length. Forty-eight (45.3%) of the novel miRNAs belong to the 24-nucleotide class, representing the most abundant novel miRNAs. 

Among the novel miRNAs, dla-miRC1 had the highest expression in our data, with 4,801 reads. Based on their frequencies and sequences in the small RNA library, although the expression levels of these candidates ranged from thousands of reads to single reads, in general, novel miRNA candidates of *D. latiflorus* showed lower expression compared with most of the conserved families. The low abundance of novel miRNAs might indicate that these miRNAs play a specific role in certain tissues or developmental stages, and may be considered young miRNAs in terms of evolution [41]. This sRNA library was only generated from leaf tissues, and future experiments will be carried out to determine whether these low-abundant miRNAs are expressed at higher levels in other organs, such as flowers and seeds, or whether they are regulated by certain stresses. 

### Prediction of miRNA targets in ma bamboo

The identification of miRNA targets using bioinformatics approaches is an essential step to further understand the regulatory function of miRNAs [25,33,55]. Given the lack of genomic data for ma bamboo, the CDS from the genome of the closely related species moso bamboo and ESTs of ma bamboo were used to predict new miRNA target genes, using the criteria described in the material and methods. We identified 176 potential targets, including 139 targets from moso bamboo in File S2 and 37 targets from ma bamboo (Table 4).

**Table 4:** Potential targets gene for novel miRNAs in *D. latiflorus* based on EST data of *D. latiflorus*.

**miRNA name**	**Target ID**	**Inhibition**	**Target annotation**
dla-miRC1	JK012907.1	Cleavage	auxin response factor 15
	JK010246.1	Cleavage	auxin response factor
dla-miRC2	JK012063.1	Cleavage	phospholipase D
dla-miRC4	JK015107.1	Cleavage	phosphoribosyl transferase
dla-miRC5	JK010514.1	Translation	R3H domain containing protein
	JK015560.1	Translation	BRASSINOSTEROID INSENSITIVE 1-associated receptor kinase 1 precursor
	JK008104.1	Cleavage	NLI interacting factor-like phosphatase
	JK016203.1	Translation	sialyltransferase family domain containing protein
	JK008046.1	Cleavage	expressed protein
	JK016171.1	Translation	gibberellin response modulator protein
	JK016234.1	Cleavage	expressed protein
	JK011714.1	Translation	ethylene-responsive protein related
	JK012545.1	Translation	expressed protein
	JK011067.1	Translation	glucan endo-1,3-beta-glucosidase-related
	JK015476.1	Translation	LIM domain-containing protein
	JK008460.1	Cleavage	transporter,major facilitator family
dla-miRC15	JK016083.1	Translation	Cyclopropane-fatty-acyl-phospholipid synthase
dla-miRC27-5p	JK015342.1	Translation	protein phosphatase 2C containing protein
dla-miRC27-3p	JK009988.1	Cleavage	metallo-beta-lactamase
dla-miRC31	JK014807.1	Translation	protein kinase family protein
dla-miRC33	JK009785.1	Cleavage	ATP synthase B chain, chloroplast precursor
	JK013093.1	Cleavage	RNA recognition motif containing protein
	JK008661.1	Cleavage	expressed protein
	JK008474.1	Cleavage	SCARECROW
dla-miRC35	JK010448.1	Cleavage	CBS domain containing membrane protein
	JK012636.1	Cleavage	CBS domain containing membrane protein
	JK014547.1	Cleavage	CBS domain containing membrane protein
	JK016367.1	Cleavage	CBS domain containing membrane protein
	JK016562.1	Cleavage	CBS domain containing membrane protein
	JK011948.1	Cleavage	RNA polymerase sigma factor
	JK012079.1	Cleavage	RNA polymerase sigma factor
	JK012720.1	Cleavage	RNA polymerase sigma factor
	JK013456.1	Cleavage	RNA polymerase sigma factor
	JK012868.1	Cleavage	RNA polymerase sigma factor
	JK016358.1	Cleavage	RNA polymerase sigma factor
	JK014679.1	Cleavage	RNA polymerase sigma factor
dla-miRC50	JK012577.1	Translation	expressed protein

Among the novel miRNAs, dla-miRC5 has 26 putative target genes with different functions, which indicates that dla-miRC5 might be involved in regulating the expression of multiple genes in ma bamboo. Some of the novel miRNAs target transcription factor (TF) genes that have been confirmed to play key roles in plant development. The targets of dla-miRC1 are auxin response factor (ARF) TF genes. ARF can regulate auxin response genes by binding specifically to *cis*-elements of their promoters to affect the developmental process of plants [55]. To date, miR160 and miR167 have been demonstrated to inhibit ARF10, ARF16, ARF17 [8,56] and ARF6, ARF8 [57], respectively; however, dla-miRC1 is barely homologous to miR160 and miR167, indicating that it might be another specific suppressor of ARF genes in ma bamboo. 

In addition, an ERF gene (belonging to AP2/EREBP family) is predicted to be a target of dla-miRC37, a MYB TF gene is a putative target of dla-miRC56, and both dla-miRC9 and dla-miRC16 have one target gene belonging to the BHLH family of TFs. Scarecrow, of the GRAS TF gene family, is targeted by dla-miRC33. All the targets of these novel miRNAs were TF genes that function in regulating the development of plants [58-61], which indicated these miRNAs might play important roles in ma bamboo development and stress responses.

Some novel miRNAs, including dla-miRC2, dla-miRC5, dla-miRC13, dla-miRC35 and dla-miC45, are predicted to target genes related to chloroplast synthesis and photosynthesis in the leaf, which is the most important photosynthetic organ of plants. This result suggested that the miRNAs might be leaf-specific and be involved in the process of regulating chloroplast synthesis and photosynthesis.

### Expression profiles of novel miRNAs in response to light

The expression patterns of miRNAs could provide clues to their functions [22]. As an efficient and sensitive method for detecting gene expression [62], stem-loop qRT-PCR was developed based on the common qRT-PCR method, with advantages such as increased sensitivity and high accuracy. Therefore, stem-loop qRT-PCR has been widely employed to distinguish two miRNAs with small differences [3].

**Figure 4:**
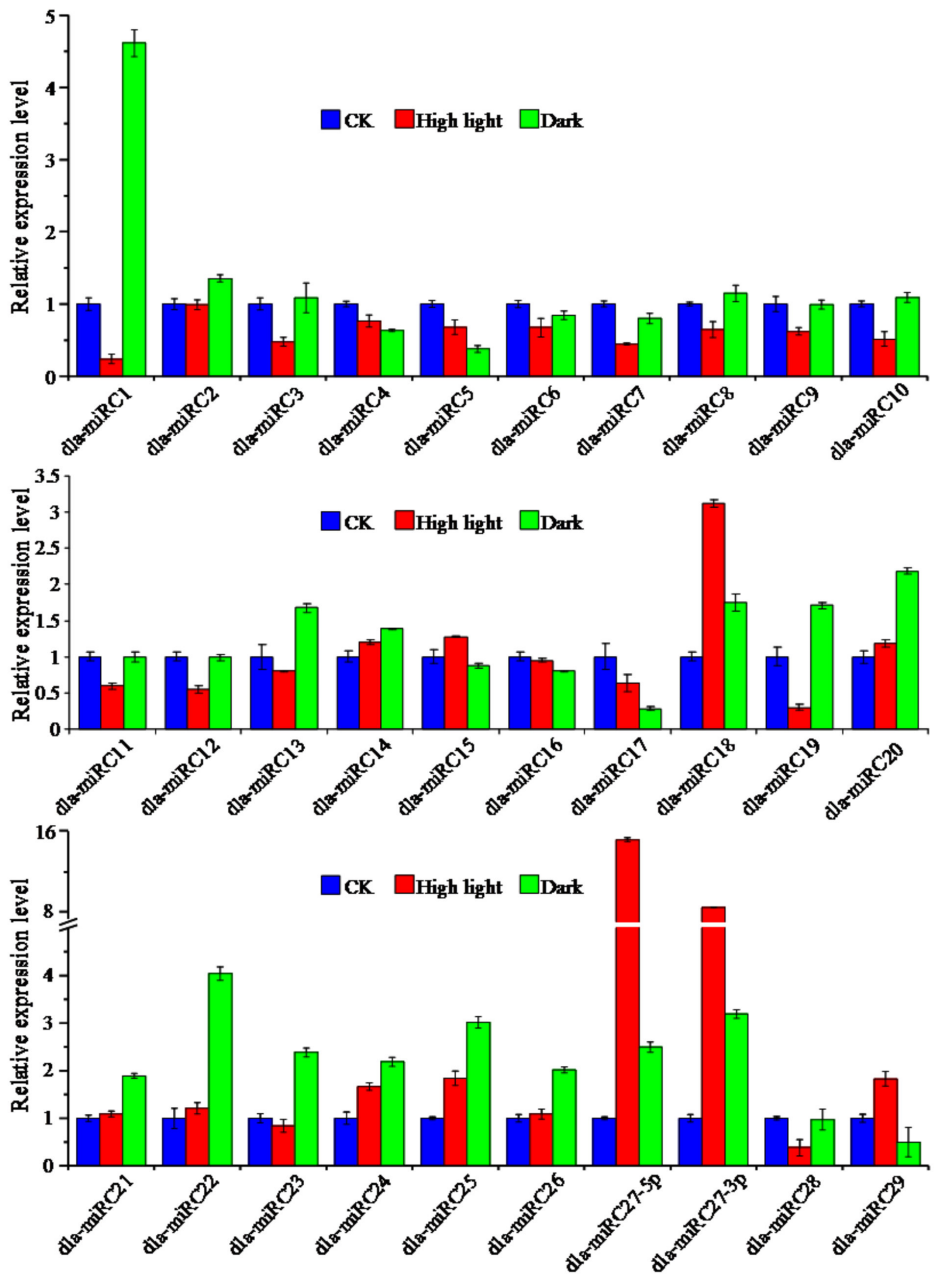
Expression profiles of novel miRNAs in leaves of *D. latiflorus* response to different light conditions. CK: 200 µmol·m^-2^·s^-1^ for 4 h; High light: 1200 µmol·m^-2^·s^-1^ for 4 h; Dark: dark for 24 h.

Among the novel predicted miRNAs, the top 30 novel miRNAs (according to the number of reads) were selected and primers were designed based on their highly specific stem-loop reverse sequences to experimentally identify their expression. As shown in File S3, the result of stem-loop qRT-PCR showed that each of the selected miRNAs had a highly specific dissolution curve, which indicated that the primers could be used for further analysis. Moreover, as shown in Figure 4, the in-depth analysis demonstrated the expression of the miRNAs under high light stress conditions; the number of miRNAs that were upregulated and downregulated was 10 and 16, respectively. The expressions of dla-miRC18, dla-miRC27-5p and dla-miRC27-3p were significantly increased under high light (P<0.01), among which dla-miRC18 was upregulated to 15 times the level of the control. However, the expression of dla-miRC1, dla-miRC19 and dla-miRC28 was significantly downregulated (P<0.01). Under dark condition, the expressions of dla-miRC1, dla-miRC22, dla-miRC25, dla-miRC27-5p and dla-miR27-3p were upregulated significantly (P<0.01), while the expressions of dla-miRC5, dla-miRC17 and dla-miRC29 were downregulated significantly (P<0.01). 

This analysis also indicated that the expressions of dla-miRC1 and dla-miRC19 were downregulated significantly under high light and upregulated markedly under dark conditions (P<0.01), indicating that they were inhibited by light. However, dla-miRC29 was upregulated under high light and downregulated under dark, indicating it was light-induced expression. MiR27-5p and miR27-3p were cleaved from the same precursor and had similar expression profiles, being upregulated under both high light and dark, which indicated they might have the same promoter and *cis*-elements, and synergistic expression characteristics. The expression results indicated that these miRNAs were affected by light, and might play vital roles in the regulation of genes involved in light signal transduction and light stress.

Bamboo converts light energy into chemical energy through photosynthesis, which is one of the necessary processes supplying carbohydrates for the rapid expansion of cells. As the first comparative identification of miRNAs among leaves treated with distinct light conditions, this analysis showed that bamboo may possess a unique light regulation mechanism involving miRNAs, although its function and mechanism are currently unknown. 

## Conclusions

We identified 81 novel miRNAs and 84 conserved miRNAs belonging to 17 families in the leaf of ma bamboo using high-throughput sequencing and bioinformatics analysis. The results of qRT-PCR indicated the miRNAs might regulate the expression of genes involved in photosynthesis, which acts as a key metabolic pathway in the fast growth of bamboo. These miRNAs will add to the growing database of new miRNAs and lay the foundation for further understanding of miRNA function in the regulation of ma bamboo development and other biological characteristics. These miRNAs identified in the leaf of ma bamboo provide new opportunities for future functional genome research in bamboo and other related species.

## Supporting Information

File S1 **Primers used in novel miRNAs qRT-PCR.**
(DOC)

File S2 **Potential targets gene for novel miRNAs in *D. latiflorus* based on moso bamboo CDS data.**
(DOC)

File S3 **Dissolution curves of U6 snRNA and 30 novel miRNAs.**
(DOC)
