# Supplementary material for: Identification and Characterization of MicroRNAs in the Leaf of Ma Bamboo (Dendrocalamus latiflorus) by Deep Sequencing
Source: PLoS One. 2013 Oct 21;8(10):e78755. doi: 10.1371/journal.pone.0078755 (PMC3804618; doi:10.1371/journal.pone.0078755)
Supplement: File S1 — Primers used in novel miRNAs qRT-PCR. (DOC) [file pone.0078755.s001.doc]

Table S1. Primers used in novel miRNAs qRT-PCR

| **Name** | **Primer** | | **Sequence** |
| --- | --- | --- | --- |
| dla-miRC1 | RT Primer | | CTCAACTGGTGTCGTGGAGTCGGCAATTCAGTTGAGTCTGGGTT |
|  | Forward Primer | | ACTCACCTCGTCTTGACCTTGTA |
| dla-miRC2 | RT Primer | | CTCAACTGGTGTCGTGGAGTCGGCAATTCAGTTGAGATGCAGTC |
|  | Forward Primer | | AATCATATCCAACCGTTGGGC |
| dla-miRC3 | RT Primer | | CTCAACTGGTGTCGTGGAGTCGGCAATTCAGTTGAGGCCTCTTT |
|  | Forward Primer | | CGAGAATTACCCGGGAGTAGA |
| dla-miRC4 | RT Primer | | CTCAACTGGTGTCGTGGAGTCGGCAATTCAGTTGAGACGTGCCC |
|  | Forward Primer | | AGTAGCATTGCTGGAGAAGCA |
| dla-miRC5 | RT Primer | | CTCAACTGGTGTCGTGGAGTCGGCAATTCAGTTGAGTCTTCTCT |
|  | Forward Primer | | ATCACAAGGGAGAGAGGAGGAG |
| dla-miRC6 | RT Primer | | CTCAACTGGTGTCGTGGAGTCGGCAATTCAGTTGAGCTTCCTAT |
|  | Forward Primer | | GACAATCGCTGGCTATAGGATAG |
| dla-miRC7 | RT Primer | | CTCAACTGGTGTCGTGGAGTCGGCAATTCAGTTGAGATTCCCGC |
|  | Forward Primer | | ACTAGTTGGCCCTGTTTGGAAT |
| dla-miRC8 | RT Primer | | CTCAACTGGTGTCGTGGAGTCGGCAATTCAGTTGAGCTCCATCT |
|  | Forward Primer | | ACTCAGAGCCAAGAACGTGTAAG |
| dla-miRC9 | RT Primer | | CTCAACTGGTGTCGTGGAGTCGGCAATTCAGTTGAGGCCCCGTT |
|  | Forward Primer | | ATATGGGTGGAGCTGTGCCA |
| dla-miRC10 | RT Primer | | CTCAACTGGTGTCGTGGAGTCGGCAATTCAGTTGAGTCTGTGAT |
|  | Forward Primer | | ACCATCACCATAGTAGACGCCTC |
| dla-miRC11 | RT Primer | | CTCAACTGGTGTCGTGGAGTCGGCAATTCAGTTGAGAGCCTTTT |
|  | Forward Primer | | ATGAGGACACCTGGGAGTAGAA |
| dla-miRC12 | RT Primer | | CTCAACTGGTGTCGTGGAGTCGGCAATTCAGTTGAGATGCAGTC |
|  | Forward Primer | | AGCACTACCAACCATTGGGC |
| dla-miRC13 | RT Primer | | CTCAACTGGTGTCGTGGAGTCGGCAATTCAGTTGAGTGAAATTC |
|  | Forward Primer | | GTCTCGCCGTTTGGAACATAG |
| dla-miRC14 | RT Primer | | CTCAACTGGTGTCGTGGAGTCGGCAATTCAGTTGAGAACCGTCT |
|  | Forward Primer | | ACGGAGCCGATACTAGAATATCAC |
| dla-miRC15 | RT Primer | | CTCAACTGGTGTCGTGGAGTCGGCAATTCAGTTGAGCGCTTCCT |
|  | Forward Primer | | ACTGTCCGTCAGGAAGAGAGAGA |
| dla-miRC16 | RT Primer | | CTCAACTGGTGTCGTGGAGTCGGCAATTCAGTTGAGCCTGTTTG |
|  | Forward Primer | | AATGACTGGTGGAGCTGTGC |
| dla-miRC17 | RT Primer | | CTCAACTGGTGTCGTGGAGTCGGCAATTCAGTTGAGCCGTTTGG |
|  | Forward Primer | | ACTTCGGCAGGTAGAGCTGTG |
| dla-miRC18 | RT Primer | | CTCAACTGGTGTCGTGGAGTCGGCAATTCAGTTGAGACACCTCT |
|  | Forward Primer | | ACCACTGGTTCGGTCCATGT |
| dla-miRC19 | RT Primer | | CTCAACTGGTGTCGTGGAGTCGGCAATTCAGTTGAGACCATGTG |
|  | Forward Primer | | ACCACTTGGACTGACCAAGC |
| dla-miRC20 | RT Primer | | CTCAACTGGTGTCGTGGAGTCGGCAATTCAGTTGAGATCACAGA |
|  | Forward Primer | | ACCGACCTTCTAGATAAGAACCG |
| dla-miRC21 | RT Primer | | CTCAACTGGTGTCGTGGAGTCGGCAATTCAGTTGAGCCGAGGTC |
|  | Forward Primer | | AATAAGGCCCTGACTGCGTC |
| dla-miRC22 | RT Primer | | CTCAACTGGTGTCGTGGAGTCGGCAATTCAGTTGAGAATTTTTC |
|  | Forward Primer | | ACATAGGACGCAGGAATCGG |
| dla-miRC23 | RT Primer | | CTCAACTGGTGTCGTGGAGTCGGCAATTCAGTTGAGTCCCTCCC |
|  | Forward Primer | | AGCCACCTAGGACCAAGAGG |
| dla-miRC24 | RT Primer | | CTCAACTGGTGTCGTGGAGTCGGCAATTCAGTTGAGTTTGGTTT |
|  | Forward Primer | | GACGCAACACAGACGGTCTCT |
| dla-miRC25 | RT Primer | | CTCAACTGGTGTCGTGGAGTCGGCAATTCAGTTGAGATGCCAGC |
|  | Forward Primer | | AGGCTCGTATGAAGGTTTGTTG |
| dla-miRC26 | RT Primer | | CTCAACTGGTGTCGTGGAGTCGGCAATTCAGTTGAGACTATTCT |
|  | Forward Primer | | AGGAGAGGCAAGTGCATGGT |
| dla-miRC27-5p | RT Primer | | CTCAACTGGTGTCGTGGAGTCGGCAATTCAGTTGAGACGATCTT |
|  | Forward Primer | | AGCCGTCATCATAGCCACAA |
| dla-miRC27-3p | RT Primer | | CTCAACTGGTGTCGTGGAGTCGGCAATTCAGTTGAGATGTCATC |
|  | Forward Primer | | CCGTCGATCTTCTGTGGCTAT |
| dla-miRC28 | RT Primer | | CTCAACTGGTGTCGTGGAGTCGGCAATTCAGTTGAGCTAATTAC |
|  | Forward Primer | | ACGCTACACTCACAGACGGTT |
| dla-miRC29 | RT Primer | | CTCAACTGGTGTCGTGGAGTCGGCAATTCAGTTGAGCCGTTTGG |
|  | Forward Primer | | ACACGTCGCACTAGAGCTGTG |
|  |  | |  |
| Universal Reverse Primer | | | CTCAACTGGTGTCGTGGAGTC |
|  | | |  |
| U6 | | Forward Primer | GGACATCCGATAAAATTGGAACGATACAG |
|  |  | Reverse Primer | AATTTGGACCATTTCTCGATTTATGCGTGT |
