# Supplementary material for: Identification and Characterization of MicroRNAs in the Leaf of Ma Bamboo (Dendrocalamus latiflorus) by Deep Sequencing
Source: PLoS One. 2013 Oct 21;8(10):e78755. doi: 10.1371/journal.pone.0078755 (PMC3804618; doi:10.1371/journal.pone.0078755)
Supplement: File S2 — Potential targets gene for novel miRNAs in D. latiflorus based on moso bamboo CDS data. (DOC) [file pone.0078755.s002.doc]

Table S2. Potential targets gene for novel miRNAs in *D. latiflorus* based on moso bamboo CDS data

| **miRNA name** | **Target ID** | **Inhibition** | **Target annotation** |
| --- | --- | --- | --- |
| dla-miRC1 | PH01001979G0180 | Cleavage | tubulin/FtsZ domain containing protein |
|  | PH01001979G0180 | Cleavage | tubulin/FtsZ domain containing protein |
|  | PH01000237G0420 | Cleavage | Auxin response factor (ARF) 14 |
|  | PH01000018G0940 | Cleavage | Auxin response factor (ARF) |
|  | PH01001690G0310 | Cleavage | Auxin response factor (ARF) |
|  | PH01001690G0310 | Cleavage | Auxin response factor (ARF) |
|  | PH01000046G0220 | Cleavage | Auxin response factor (ARF) 15 |
|  | PH01000046G0220 | Cleavage | Auxin response factor (ARF) 15 |
|  | PH01002160G0180 | Cleavage | Auxin response factor (ARF) |
|  | PH01000002G3110 | Cleavage | Auxin response factor (ARF) |
|  | PH01001716G0120 | Cleavage | expressed_protein |
|  | PH01001727G0310 | Cleavage | PPR repeat containing protein |
| dla-miRC2 | PH01002521G0110 | Translation | acyl-desaturase, chloroplast precursor |
|  | PH01000178G0320 | Translation | expressed_protein |
| dla-miRC4 | PH01000183G1320 | Cleavage | No apical meristem (NAM) protein |
|  | PH01000110G0680 | Cleavage | No apical meristem (NAM) protein |
|  | PH01000093G0340 | Cleavage | No apical meristem (NAM) protein |
|  | PH01000501G0450 | Cleavage | No apical meristem (NAM) protein |
|  | PH01000041G2170 | Cleavage | No apical meristem (NAM) protein |
|  | PH01001309G0120 | Cleavage | No apical meristem (NAM) protein |
|  | PH01000483G1000 | Cleavage | No apical meristem (NAM) protein |
|  | PH01001391G0220 | Cleavage | expressed_protein |
|  | PH01001175G0200 | Cleavage | boron transporter protein |
| dla-miRC5 | PH01000835G0430 | Cleavage | expressed_protein |
|  | PH01000242G1390 | Cleavage | AP2 domain containing protein |
|  | PH01012023G0010 | Translation | expressed_protein |
|  | PH01000437G0690 | Cleavage | phenylalanyl-tRNA synthetase |
|  | PH01000216G0590 | Cleavage | expressed_protein |
|  | PH01000356G1120 | Translation | CBS domain containing membrane protein |
|  | PH01004134G0130 | Cleavage | glucan endo-1,3-beta-glucosidase-related |
|  | PH01000024G0850 | Cleavage | expressed_protein |
|  | PH01000019G1090 | Cleavage | peptidase family C78 domain containing protein |
|  | PH01004008G0180 | Cleavage | mTERF family protein, expressed |
|  | PH01004584G0090 | Cleavage | alpha-galactosidase precursor |
|  | PH01000333G1160 | Translation | psbP-related thylakoid lumenal protein 4, |
|  | PH01000773G0140 | Translation | sialyltransferase family domain containing protein |
|  | PH01001646G0400 | Cleavage | uncharacterized protein ycf45 |
| dla-miRC7 | PH01002311G0210 | Cleavage | B3 DNA binding domain containing protein |
| dla-miRC8 | PH01000216G0150 | Translation | GDSL-like lipase/acylhydrolase |
|  | PH01000441G0440 | Cleavage | phosphate/phosphoenolpyruvate translocator-related protein |
| dla-miRC9 | PH01000035G1890 | Cleavage | SAM domain containing protein |
|  | PH01002627G0250 | Cleavage | BHLH transcription factor |
| dla-miRC10 | PH01000649G0080 | Translation | expressed_protein |
|  | PH01002327G0270 | Cleavage | viral A-type inclusion protein repeat |
|  | PH01001246G0080 | Translation | WD domain, G-beta repeat domain containing protein |
|  | PH01004289G0050 | Translation | WD domain, G-beta repeat domain containing protein |
| dla-miRC13 | PH01003185G0010 | Cleavage | expressed_protein |
|  | PH01000014G2480 | Cleavage | cyclic nucleotide-gated ion channel |
|  | PH01000517G0450 | Cleavage | cyclic nucleotide-gated ion channel |
|  | PH01001326G0560 | Cleavage | SAM domain family protein, expressed |
|  | PH01000298G0450 | Cleavage | zinc finger, C3HC4 type domain containing protein |
|  | PH01003704G0210 | Translation | DNA gyrase subunit A, chloroplast/mitochondrial precursor |
| dla-miRC15 | PH01000474G0570 | Translation | expressed_protein |
|  | PH01002701G0050 | Translation | CDGSH iron sulfur domain-containing protein 1 |
|  | PH01000630G0370 | Cleavage | expressed_protein |
|  | PH01002083G0430 | Translation | methyltransferase |
|  | PH01001515G0400 | Translation | elongation factor 1-gamma |
|  | PH01003023G0120 | Translation | nodulin MtN3 family protein |
|  | PH01001305G0260 | Cleavage | ABC transporter, ATP-binding protein |
|  | PH01002470G0090 | Translation | 1,4-alpha-glucan-branching enzyme |
|  | PH01001544G0370 | Translation | Cyclopropane-fatty-acyl-phospholipid synthase |
| dla-miRC16 | PH01000440G0530 | Cleavage | peptidyl-prolyl cis-trans isomerase, FKBP-type |
|  | PH01002627G0250 | Cleavage | BHLH transcription factor |
|  | PH01000136G0140 | Translation | hypothetical_protein |
| dla-miRC17 | PH01000035G1890 | Cleavage | SAM domain containing protein |
|  | PH01000646G0090 | Translation | ABC transporter, ATP-binding protein |
|  | PH01002135G0160 | Translation | ABC transporter, ATP-binding protein |
|  | PH01001305G0260 | Translation | ABC transporter, ATP-binding protein |
|  | PH01002575G0110 | Cleavage | GDSL-like lipase/acylhydrolase |
| dla-miRC18 | PH01003591G0150 | Cleavage | zinc ion binding protein |
|  | PH01001261G0140 | Cleavage | zinc ion binding protein |
|  | PH01002909G0060 | Cleavage | amidase |
|  | PH01000836G0340 | Cleavage | F-box and other domain containing protein |
|  | PH01001726G0060 | Cleavage | trehalose-6-phosphate synthase |
| dla-miRC19 | PH01000103G0260 | Cleavage | Putative Prolyl Oligopeptidase homologue |
|  | PH01001255G0050 | Cleavage | calcium-transporting ATPase, endoplasmic reticulum-type |
|  | PH01000632G0360 | Cleavage | NAK-like ser/thr protein kinase |
| dla-miRC20 | PH01000538G0560 | Cleavage | transcription repressor HOTR |
|  | PH01005147G0010 | Cleavage | RGH1A |
| dla-miRC21 | PH01002012G0310 | Cleavage | OWAK receptor-like protein kinase |
| dla-miRC22 | PH01000544G0520 | Cleavage | glucose-6-phosphate isomerase |
| dla-miRC23 | PH01000133G0540 | Cleavage | ataxin-2 related protein |
|  | PH01001450G0410 | Cleavage | miro |
| dla-miRC25 | PH01000410G0400 | Cleavage | cleavage and polyadenylation specificity factor |
|  | PH01000716G0430 | Cleavage | expressed_protein |
|  | PH01002647G0150 | Cleavage | expressed_protein |
|  | PH01000579G0100 | Cleavage | HEN1 |
| dla-miRC26 | PH01000087G1230 | Cleavage | expressed_protein |
|  | PH01001239G0360 | Cleavage | histidine-containing phosphotransfer protein |
| dla-miRC27-5p | PH01003190G0050 | Translation | expressed_protein |
|  | PH01003644G0180 | Translation | WD domain containing protein |
|  | PH01000255G0050 | Translation | cyclin, N-terminal domain containing protein |
|  | PH01000089G0180 | Translation | protein phosphatase 2C containing protein |
| dla-miRC27-3p | PH01000672G0740 | Cleavage | purine permease |
|  | PH01000589G0690 | Cleavage | succinate dehydrogenase flavoprotein subunit |
| dla-miRC29 | PH01000097G1550 | Cleavage | pentatricopeptide |
| dla-miRC32 | PH01000402G0180 | Cleavage | periplasmic beta-glucosidase precursor |
|  | PH01000631G0700 | Translation | disease resistance protein RPM1 |
|  | PH01002438G0020 | Translation | potassium transporter |
| dla-miRC33 | PH01000169G0740 | Cleavage | extracellular ligand-gated ion channel |
|  | PH01001563G0300 | Cleavage | Putative Type I Signal Peptidase homologue; employs a putative Ser/His catalytic dyad |
|  | PH01004122G0150 | Cleavage | plasma membrane ATPase |
|  | PH01001050G0170 | Translation | expressed_protein |
|  | PH01000841G0170 | Cleavage | glycoprotein |
|  | PH01000171G1050 | Cleavage | glycoprotein |
|  | PH01001517G0310 | Cleavage | expressed_protein |
|  | PH01002771G0230 | Cleavage | RNA recognition motif containing protein |
|  | PH01002279G0010 | Cleavage | TENA/THI-4 family protein |
|  | PH01000414G0290 | Cleavage | expressed_protein |
|  | PH01000670G0270 | Cleavage | spotted leaf 11 |
|  | PH01006110G0020 | Cleavage | expressed_protein |
| dla-miRC35 | PH01000523G0830 | Cleavage | AGAP007115-PA |
|  | PH01000343G0330 | Translation | ethylene-responsive transcription factor |
|  | PH01001761G0100 | Translation | DHHC zinc finger domain containing protein |
|  | PH01000340G0740 | Cleavage | BTBA4 - Bric-a-Brac,Tramtrack, Broad Complex BTB domain with Ankyrin repeat region |
|  | PH01001801G0160 | Translation | zinc finger/CCCH transcription factor |
|  | PH01002491G0170 | Translation | Leucine Rich Repeat family protein |
|  | PH01002513G0070 | Cleavage | 1,3-beta-glucan synthase component domain |
|  | PH01001945G0320 | Translation | CSLF3 - cellulose synthase-like family F; beta1,3;1,4 glucan synthase |
| dla-miRC38-1-5p | PH01001009G0700 | Cleavage | kelch repeat protein |
|  | PH01000054G0330 | Cleavage | laccase precursor protein |
| dla-miRC38-1-3p | PH01001990G0360 | Cleavage | PHS1 |
| dla-miRC41 | PH01001672G0190 | Cleavage | hypothetical_protein |
| dla-miRC42 | PH01003704G0210 | Cleavage | DNA gyrase subunit A, chloroplast/mitochondrial precursor |
| dla-miRC50 | PH01001560G0360 | Translation | armadillo/beta-catenin repeat family protein |
| dla-miRC52 | PH01000358G0250 | Translation | Myb transcription factor |
|  | PH01000743G0310 | Cleavage | expressed_protein |
|  | PH01001750G0010 | Cleavage | structural constituent of ribosome |
| dla-miRC53 | PH01005219G0020 | Cleavage | CAAX amino terminal protease family protein |
|  | PH01000669G0280 | Translation | eukaryotic peptide chain release factor subunit |
|  | PH01000370G1160 | Cleavage | ribonuclease protein |
|  | PH01001939G0120 | Translation | gar2 |
| dla-miRC56 | PH01001901G0020 | Cleavage | GTP binding protein |
| dla-miRC60 | PH01001042G0090 | Cleavage | Superfamily of TFs having WRKY and zinc finger domains |
|  | PH01000029G2170 | Cleavage | EAP30/Vps36 family domain containing protein |
| dla-miRC61 | PH01002913G0170 | Cleavage | hypothetical_protein |
|  | PH01002119G0110 | Cleavage | pentatricopeptide repeat domain |
| dla-miRC62 | PH01000145G0570 | Cleavage | cleavage and polyadenylation specificity factor |
|  | PH01006136G0030 | Cleavage | ubiquitin carboxyl-terminal hydrolase domain |
