# Supplementary figures and images for: Identification and Characterization of MicroRNAs in the Leaf of Ma Bamboo (Dendrocalamus latiflorus) by Deep Sequencing
Source: PLoS One. 2013 Oct 21;8(10):e78755. doi: 10.1371/journal.pone.0078755 (PMC3804618; doi:10.1371/journal.pone.0078755)

Figure S1. Dissolution curves of U6 snRNA and 30 novel miRNAs


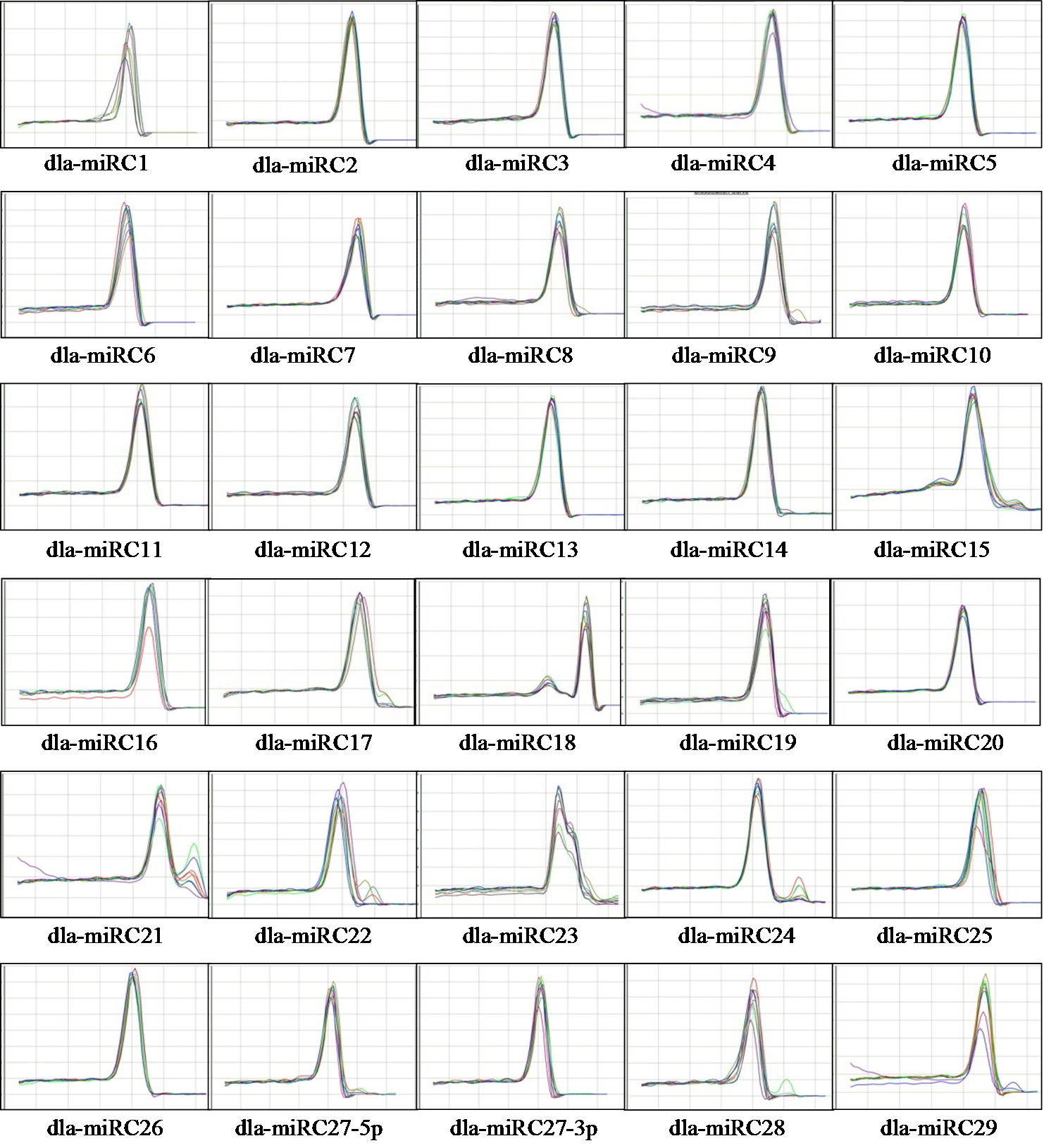


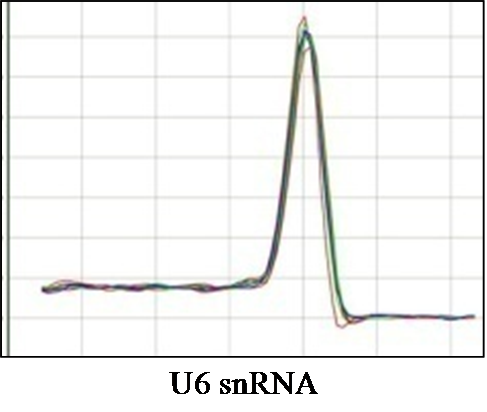

Supplement: File S3 — Dissolution curves of U6 snRNA and 30 novel miRNAs. (DOC) [file pone.0078755.s003.doc]
